# Multimodal analysis suggests differential immuno-metabolic crosstalk in lung squamous cell carcinoma and adenocarcinoma

**DOI:** 10.1038/s41698-021-00248-2

**Published:** 2022-01-27

**Authors:** Brooks P. Leitner, Kevin B. Givechian, Shyryn Ospanova, Aray Beisenbayeva, Katerina Politi, Rachel J. Perry

**Affiliations:** 1 Department of Cellular & Molecular Physiology, Yale School of Medicine, New Haven, CT USA; 2 Department of Internal Medicine (Endocrinology), Yale School of Medicine, New Haven, CT USA; 3 Department of Genetics, Yale School of Medicine, New Haven, CT USA; 4 Nazarbayev Intellectual School of Physics and Mathematics, Almaty, Kazakhstan; 5 Kazakhstan International School, Almaty, Kazakhstan; 6 Department of Pathology, Yale School of Medicine, New Haven, CT USA; 7 Department of Internal Medicine (Oncology), Yale School of Medicine, New Haven, CT USA; 8 Yale Cancer Center, Yale School of Medicine, New Haven, CT USA

**Keywords:** Cancer imaging, Cancer genomics, Non-small-cell lung cancer, Cancer metabolism

## Abstract

Immunometabolism within the tumor microenvironment is an appealing target for precision therapy approaches in lung cancer. Interestingly, obesity confers an improved response to immune checkpoint inhibition in non-small cell lung cancer (NSCLC), suggesting intriguing relationships between systemic metabolism and the immunometabolic environment in lung tumors. We hypothesized that visceral fat and ^18^F-Fluorodeoxyglucose uptake influenced the tumor immunometabolic environment and that these bidirectional relationships differ in NSCLC subtypes, lung adenocarcinoma (LUAD) and lung squamous cell carcinoma (LUSC). By integrating ^18^F-FDG PET/CT imaging, bulk and single-cell RNA-sequencing, and histology, we observed that LUSC had a greater dependence on glucose than LUAD. In LUAD tumors with high glucose uptake, glutaminase was downregulated, suggesting a tradeoff between glucose and glutamine metabolism, while in LUSC tumors with high glucose uptake, genes related to fatty acid and amino acid metabolism were also increased. We found that tumor-infiltrating T cells had the highest expression of glutaminase, ribosomal protein 37, and cystathionine gamma-lyase in NSCLC, highlighting the metabolic flexibility of this cell type. Further, we demonstrate that visceral adiposity, but not body mass index (BMI), was positively associated with tumor glucose uptake in LUAD and that patients with high BMI had favorable prognostic transcriptional profiles, while tumors of patients with high visceral fat had poor prognostic gene expression. We posit that metabolic adjunct therapy may be more successful in LUSC rather than LUAD due to LUAD’s metabolic flexibility and that visceral adiposity, not BMI alone, should be considered when developing precision medicine approaches for the treatment of NSCLC.

## Introduction

Lung cancer is one of the few tumor types that are not commonly associated with systemic metabolic dysregulation. Clinically, it has not been associated with excess body weight and obesity by the U.S. Centers for Disease Control^1^, and interestingly, several reports have suggested that excess body weight may actually reduce the risk and slow the progression of lung cancer^2–6^ especially in those treated with immune checkpoint inhibitors^7,8^. However, studies of how body composition and tumor metabolism affect lung cancer outcomes have rarely differentiated between the subtypes of lung cancer, and even more rarely have differentiated between the subtypes of non-small cell lung cancer (NSCLC). This knowledge gap limits the possibilities of developing metabolic strategies to combat lung cancer using a precision medicine approach.

Tumor glucose uptake, most commonly measured by positron emission tomography–computed tomography (PET-CT) with [^18^F]-fluorodeoxyglucose (^18^F-FDG) in humans, has long been utilized as a marker of metabolic activity. Glucose taken up by tumors primarily fuels glycolytic metabolism, which produces glucose- and fructose-6-phosphate and, in turn, generates the cellular building blocks (nucleotides, macromolecules) needed for rapid cell division. Therefore, increased tumor glucose uptake is typically a poor prognostic marker. We recently demonstrated in an analysis of PET-CT images from The Cancer Imaging Archive (TCIA) that body mass index (BMI) correlated negatively with the lean body mass-corrected maximum standard uptake value (SUVmax) in NSCLC^9^, consistent with the prior epidemiologic data. Yet, recent data in NSCLC has suggested that ^18^F-FDG does not correlate with glycolytic capacity per se but more closely with proliferation index^10^, begging both a deeper and more comprehensive analysis of the metabolic pathways related to NSCLC nutrient metabolism.

Though BMI is a powerful tool for epidemiological research, normal, overweight, and obese classifications oversimplify the potential impact of weight on metabolic health. Athletes may be overweight with good metabolic health, while certain populations including older adults^11–13^ and individuals of South and East Asian descent^14,15^, may have poor lean muscle mass and excess adiposity despite BMIs in the healthy range. Consistent with this, previous analyses correlating anthropometric indices to visceral adipose tissue (VAT), a strong predictor of cardiometabolic risk, demonstrated that BMI did not correlate with VAT^16,17^. These data highlight the need to use markers of adiposity, rather than BMI, when examining how excess body weight affects cancer progression in order to develop targeted approaches to improve outcomes. Considering routinely collected PET-CT scans allow one to quantify skeletal muscle mass, visceral and subcutaneous adiposity, and tissue and tumor-specific glucose uptake, we leveraged one of the largest open-access nuclear medicine imaging databases, TCIA, to extract imaging-related features to move beyond BMI, an oversimplified surrogate for body composition.

Given that recent evidence suggests that PET glucose uptake may reflect immune cell metabolism rather than tumor cells^18^, we examined tumor-infiltrating leukocytes (TILs) identified by caMicroscope and validated by pathologists in the tumors of patients with available PET/CT image data. In addition, RNA-sequencing has proven to reveal metabolic vulnerabilities through transcriptomic analyses of the tumor microenvironment^19^, so we employed The Cancer Genome Atlas (TCGA) analyses to gain a deeper understanding of the metabolic transcriptomic tumor landscape and ultimately advance toward identifying metabolic pathways relevant to the development of new metabolism-targeting approaches for precision oncology. With the explosion of single-cell RNA-sequencing (scRNA-seq), even more insight into cell-type-specific metabolic crosstalk has become possible; using genes identified based on PET-defined glucose uptake, we explored which tumor-infiltrating cells may have a unique capacity for metabolic flexibility.

A unique aspect of the current study is the comparison between the two most common NSCLC types, lung adenocarcinoma (LUAD) and lung squamous cell carcinoma (LUSC), which comprises 85% of all lung cancer cases. A recent analysis of data from >37,000 lung cancer patients found differences in demographics and in outcomes: LUSC patients tend to be older, more likely male and more likely smokers^20^. First-line treatment protocols of these subtypes differ, and results regarding relative survival rates between these NSCLC subtypes vary, with improved survival in LUSC^21–24^ and LUAD^20,25–28^ having both been reported. Further, recent genomic analyses suggest differences in immunogenicity between LUSC and LUAD^29^.

In this study, we employed a multimodal approach to understanding how body composition, metabolism, and immune cell composition intersect with tumor glucose uptake, and tumor genomics in NSCLC. We hypothesized that features derived from routine nuclear medicine imaging could provide unique insights into the tumor immunometabolic microenvironment. Our analyses revealed specific metabolic signatures in LUSC vs LUAD and derived new correlations between immune cell subpopulations, body composition, tumor glucose uptake, and overall survival in NSCLC. We confirmed prior epidemiological evidence that patients with high BMI may have a favorable prognosis but added evidence that visceral adiposity, not excess weight, still has a deleterious transcriptional profile in lung cancer. In addition, we stratified patients with high vs low PET-derived glucose uptake for each tumor subtype. We found that differentially expressed amino acid metabolism genes differ in LUAD and LUSC. In LUAD there may be a tradeoff between glutamine metabolism and glucose metabolism in tumors and tumor-infiltrating T cells, while in LUSC, glucose metabolism is positively correlated with amino acid metabolism and biosynthetic gene transcripts, consistent with the idea that glucose fuels protein biosynthesis in tumors and T cells. We anticipate that these results could inform new precision medicine approaches to understand how body composition and glucose uptake may alter tumor genomics and metabolism in lung cancer.

## Results

### Patient characteristics

**Fig. 1:**
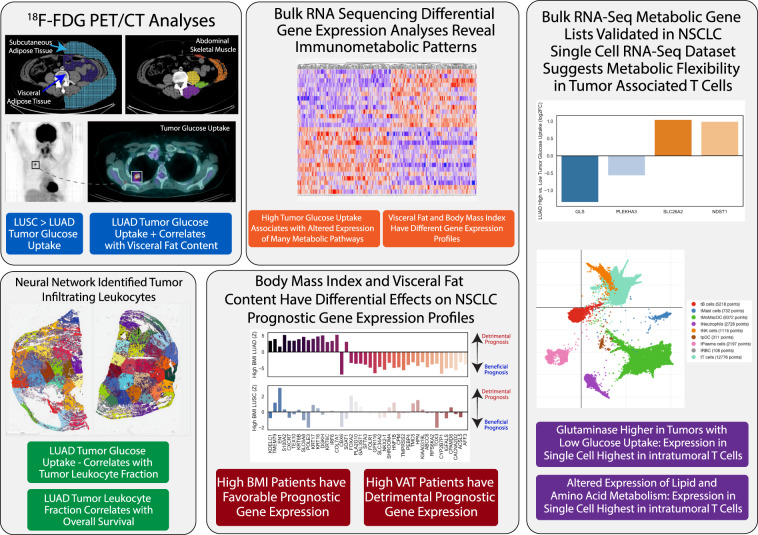
Overview of a multimodal approach to immunometabolic phenotyping of non-small cell lung cancer. Each panel shows each multimodal analysis with a representative image and key findings beneath. In clockwise direction: PET/CT imaging analyses, bulk RNA seq analyses, single cell RNA seq analyses, prognostic gene expression profiling using PRECOG database, and neural network classified histopathology.

CT-defined visceral and subcutaneous adipose tissue and tissue-specific glucose uptake by ^18^FDG-PET, tumor RNA sequencing, and histological assessment of TIL counts and subtypes were assessed (Fig. 1). Comparing the two tumor types, patients’ weight, sex, race, and tumor stage were comparable (Supplemental Table 1), although subjects were limited to 17 and 18 per tumor type due to the limited numbers of PET/CT images available in the TCIA database.

### Visceral adiposity and BMI differentially affect tumor gene expression in NSCLC

**Fig. 2:**
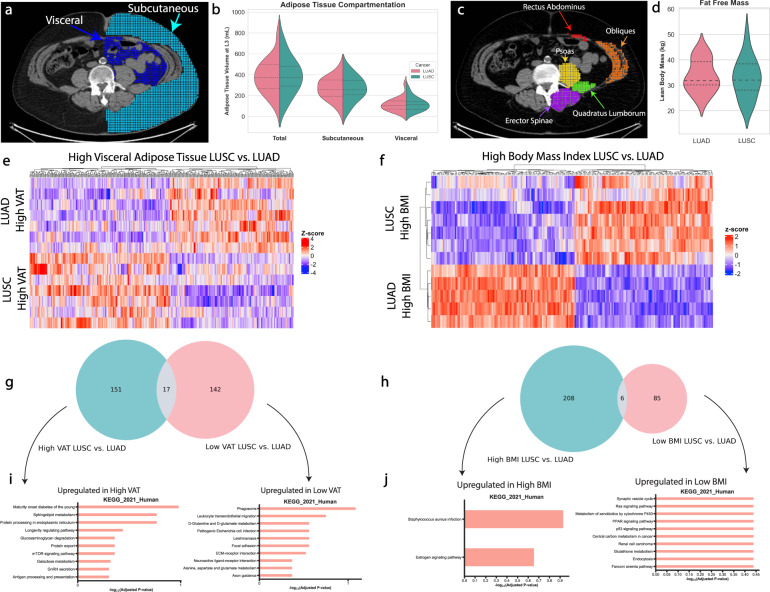
Visceral and subcutaneous adiposity differentially affect tumor gene expression in NSCLC. **a**, **b** Visceral and subcutaneous adipose tissue was quantified from CT scans from the L3–L4 vertebral level in all patients. **c**, **d** Abdominal skeletal muscle volume was quantified as the sum of five muscle groups at the L3–L4 vertebral level in all patients. Differential gene expression analyses were performed between LUAD and LUSC cohorts based on being in the top half of (**e**) VAT volume and (**f**) BMI. Venn diagrams were created to isolate genes unrelated to differences solely due to tumor subtype (**g**, **h**). Upregulated KEGG pathways were analyzed in each non-overlapping gene list (**i**, **j**). *n* = 15 for LUSC and *n* = 13 for LUAD.

We quantified visceral (VAT) and subcutaneous (SubQ) adipose tissue volume by PET-CT and found it to be comparable between subjects with LUAD and LUSC tumors (Fig. 2a, b), as in line with the identical body weight between patients with the two tumor types. Lean body mass was similar between the tumor types (Fig. 2c, d). Differential gene expression analyses revealed widespread differences when comparing patients with high VAT content and high BMI in LUSC and LUAD (Fig. 2e, f). In order to exclude genes that may have differed solely due to differences in each NSCLC tumor type, we also performed these analyses in the low VAT and low BMI patients plotted in a Venn diagram (Fig. 2g, h). Upregulated gene expression pathways in high VAT patients included several carbohydrate metabolism pathways including maturity-onset diabetes of the young, glycosaminoglycan degradation, galactose metabolism, as well as mTOR signaling. The Venn diagram in the BMI stratified patients looked drastically different from the VAT stratification (Fig. 2h), and upregulated pathways included the inflammatory and hormonal KEGG pathways, *Staphylococcus aureus* infection, and estrogen signaling (Fig. 2j).

**Fig. 3:**
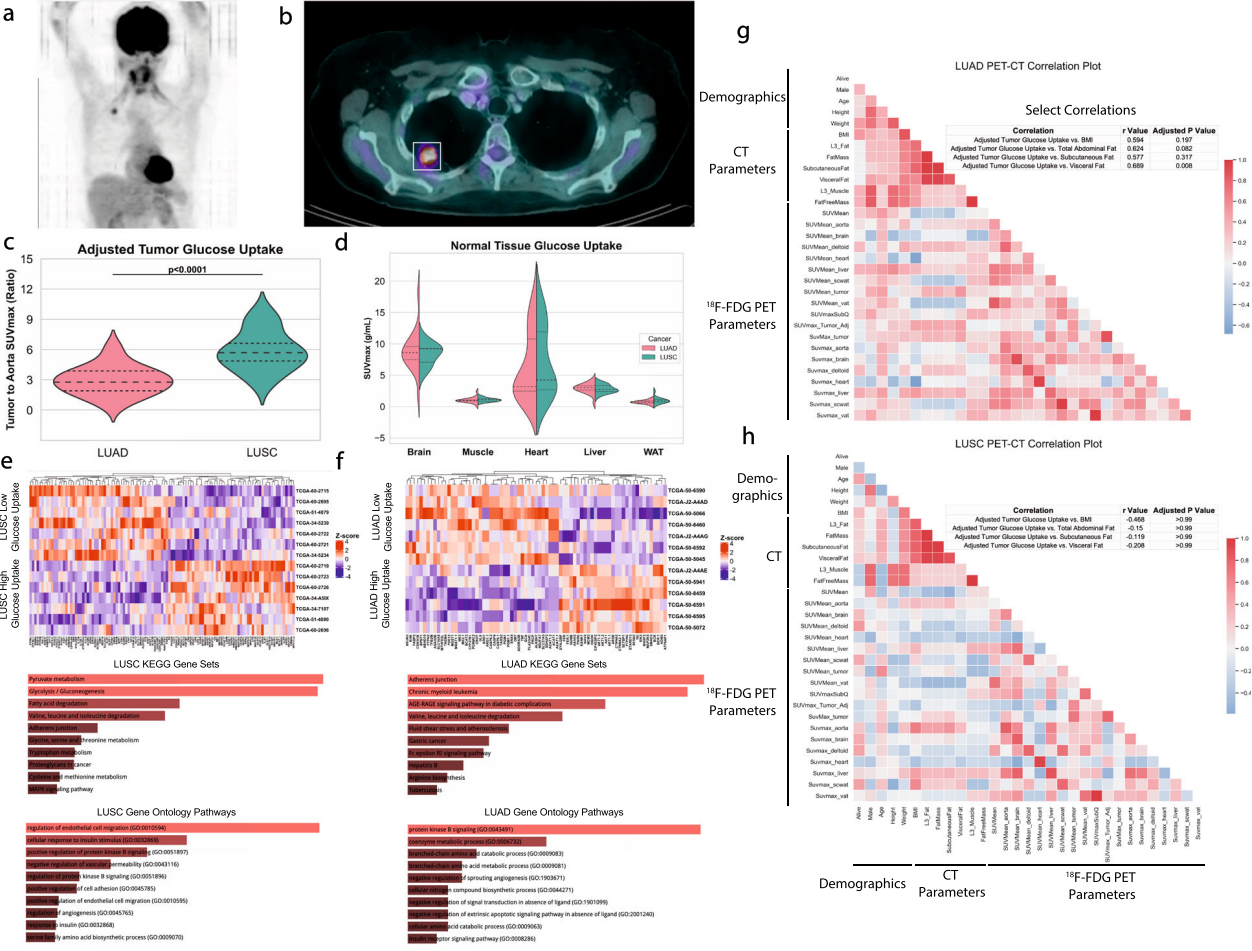
High visceral fat content is associated with a poor prognostic profile in LUAD, while high BMI is associated with a favorable prognostic profile. *Z* scores >3.09 or <−3.09 from the PRECOG database are displayed for high visceral fat (**a**), high BMI (**b**), low visceral fat (**c**), and low BMI (**d**). Positive *Z* scores have negative prognostic value while negative *Z* scores have beneficial prognostic value.

We then aimed to determine whether the prognostic value of the differentially expressed gene (DEG) expression profiles in tumors of patients with high VAT and high BMI differed (Fig. 3). *Z* scores from the PRECOG dataset were plotted, where genes with higher *Z* scores are independently associated with poorer survival in lung cancer, whereas the genes with lower *Z* scores are independently associated with better survival. LUAD patients with high VAT had a large proportion (65%) of detrimental prognostic genes, whereas the patients with high BMI leaned towards a favorable gene expression profile (62% favorable prognostic genes), consistent with epidemiological literature that high BMI is beneficial in lung cancer survival. These trends were much clearer for LUAD than LUSC, and patients with low VAT or low BMI did not demonstrate a reversal of prognostic value (Fig. 3c, d).

### The quantity and location of adipose tissue predicts differential gene expression patterns between LUAD and LUSC

**Fig. 4:**
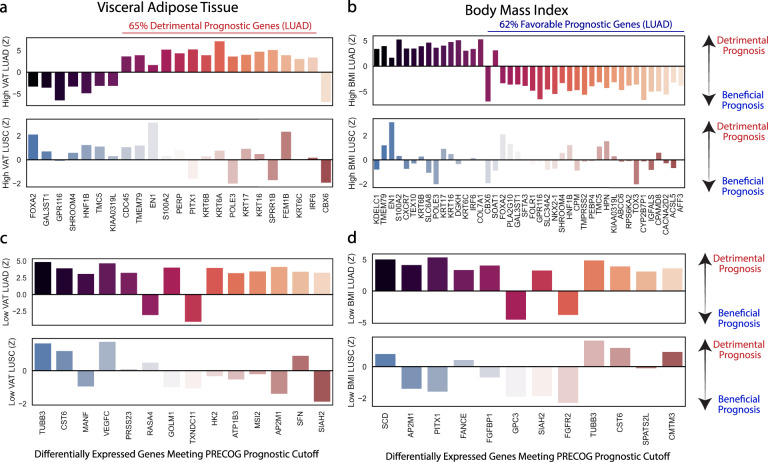
Tumor glucose uptake and metabolic genes within the tumor differ between LUAD and LUSC. **a** Representative maximal intensity projection and **b** coronal PET/CT slice identifying the lung tumor. Tumor glucose uptake was quantified as a tumor to background ratio (**c**) and as SUVmax in healthy tissues (**d**) and compared between LUAD and LUSC with a two-sided *t* test. Differential gene expression analyses with associated KEGG and GO processes for significantly upregulated or downregulated differentially expressed (*p* < 0.05) pathways were performed in **e** LUSC and **f** LUAD based on a median split within each cohort of ranked tumor glucose uptake values. Correlation matrices were performed and Pearson *r* values were calculated for demographics and nuclear medicine parameters for LUAD (**g**) and LUSC (**h**), with select hypothesis-testing correlations shown above each correlation plot. *n* = 15 for LUSC and *n* = 13 for LUAD.

Utilizing the TCIA database, we quantified tumor glucose uptake using ^18^F-FDG PET-CT and found that the SUVmax normalized to SUV in the descending aorta was onefold higher in LUSC than in LUAD (*p* < 0.001) (Fig. 4a–c), despite comparable body weight, stage, or adipose tissue or muscle volume. Consistent with a tumor-specific effect rather than a systemic effect to promote increased glucose uptake, no differences were observed in ^18^F-FDG uptake in non-tumor tissues including heart, liver, skeletal muscle (deltoid) adipose tissue (SubQ abdominal), and brain (Fig. 4d).

Having observed significantly higher glucose uptake in LUSC as compared to LUAD, we performed differential gene expression analysis on the tumors from NSCLC patients with high vs low tumor glucose uptake. This analysis revealed numerous differentially expressed KEGG metabolic pathways including pyruvate, glucose, amino acid, and fatty acid metabolism, as well as GO biological processes including angiogenesis and insulin signaling processes in LUSC (Fig. 4e). In contrast, LUAD gene expression showed a less clear association between tumor glucose uptake and metabolic gene expression, while expression of genes in angiogenesis GO pathways did correlate with tumor glucose uptake in LUAD (Fig. 4f).

Next, we aimed to determine whether body composition correlated with tumor glucose uptake within each tumor type. In general, LUAD showed more positive correlations between CT and ^18^F-FDG PET parameters than LUSC (Fig. 4g, h). Upon further interrogation, we observed that only the VAT compartment, rather than subcutaneous fat or BMI, was significantly correlated with tumor glucose uptake in LUAD (*p* = 0.008) (Fig. 4g). Further, though LUSC glucose uptake was higher than LUAD, there appeared to be no significant influence of body composition or adiposity on tumor glucose uptake in patients with LUSC (Fig. 4h).

### TILs correlate with tumor glucose uptake in both NSCLC tumor types

**Fig. 5:**
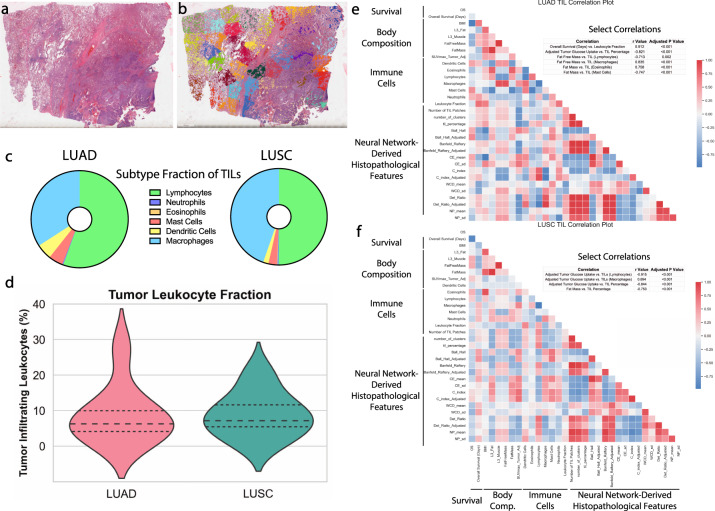
Tumor-infiltrating leukocytes are negatively correlated with tumor glucose uptake and visceral fat content in LUSC and LUAD. A representative histological tumor slice with **a** H&E only or **b** neural-network-derived clusters identifying different immune cell populations within the tumor slide. **c** Subtyping of the tumor-infiltrating leukocytes (TILs) in LUAD and LUSC tumors. **d** Proportion of tumor cells that were identified as TILs in both cohorts. Correlation matrices were performed and Pearson *r* values were calculated for survival, body composition, and TIL parameters for LUAD (**e**) and LUSC (**f**), with select hypothesis-testing correlations shown above each correlation plot. *n* = 11 for LUSC and *n* = 8 for LUAD.

TCGA database provides unique access to data on TIL density and cell type, with TIL clusters identified via a pathologist-confirmed convolutional neural network (Fig. 5a, b)^30^. Though we did not observe any differences in TIL fractions between the two subtypes, we aimed to examine whether any nuclear medicine features could provide insight into the immune cell subfraction in LUSC and LUAD (Fig. 5c–f). In both tumor types, the TIL fraction of the tumor was negatively correlated with adjusted tumor glucose uptake (*p*s < 0.001). In LUAD, the fraction of leukocytes in the tumor was significantly related to overall survival (*p* < 0.001). Further, fat-free mass was positively associated with macrophage content in LUAD tumors (*p* < 0.001), and negatively associated with lymphocyte content in LUSC tumors (*p* = 0.002). In the high glucose uptake NSCLC tumor type, LUSC, we observed that tumor glucose uptake was negatively associated with total TIL content (*p* < 0.001) and lymphocyte content (*p* < 0.001), but positively associated with macrophage content (*p* < 0.001), consistent with the recent results that macrophages consume more glucose than lymphocytes in the tumor microenvironment^18^, and suggesting that different immune cell subtypes may be differentially influenced by the local immunometabolic tumor milieu.

### scRNA-seq validation reveals T cell metabolic flexibility in the NSCLC tumor microenvironment

**Fig. 6:**
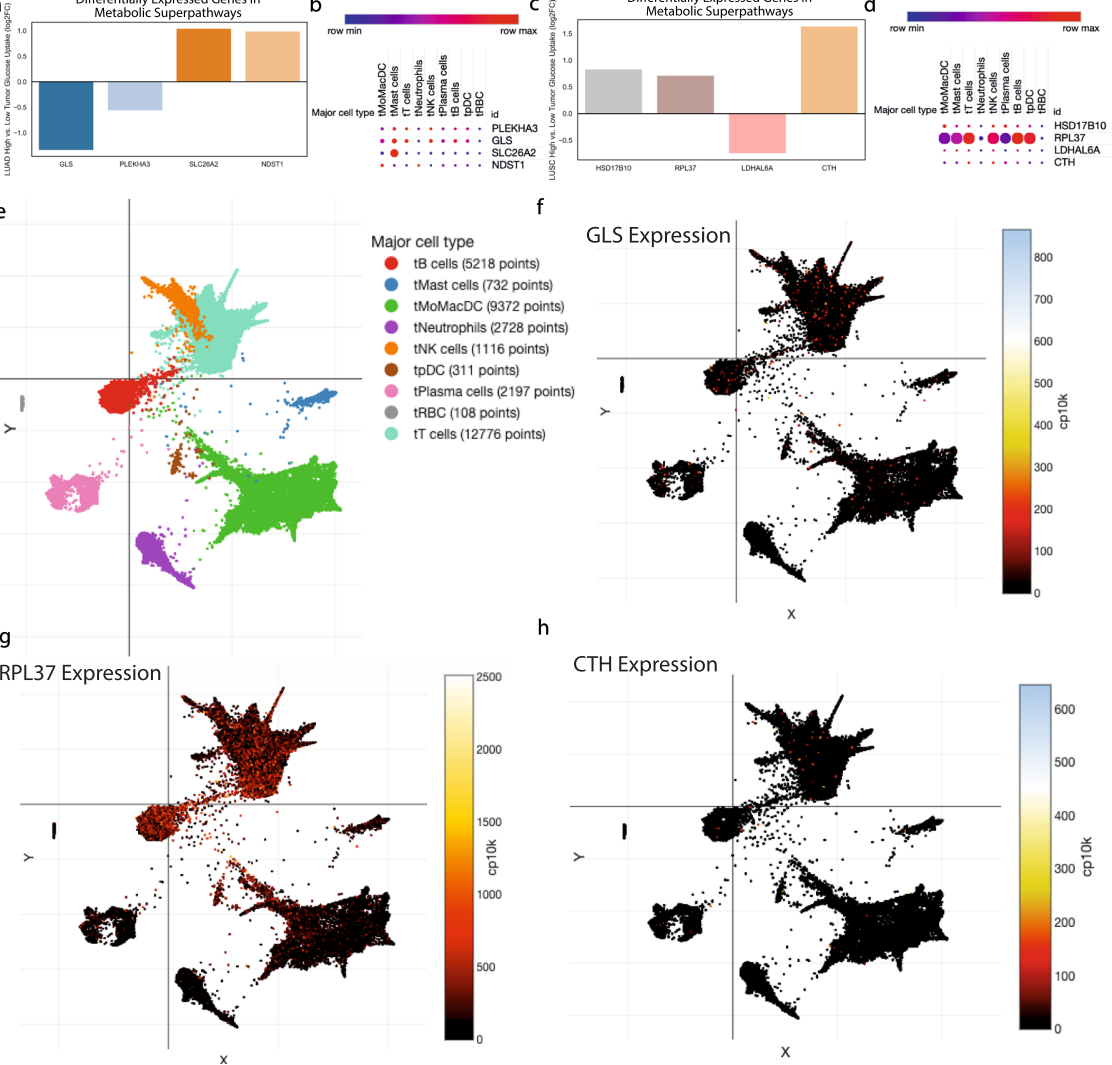
Glucose and alternative substrate metabolism differ in LUAD and LUSC, in part due to tumor-infiltrating T cell metabolic flexibility. Differentially expressed genes based on high vs low tumor glucose uptake in each tumor type were examined in a NSCLC single-cell RNA-seq database to examine tumor-infiltrating cell-type-specific expression (**a**–**d**). Data reduction analysis defined all major tumor-infiltrating subtypes (**e**), while three genes of interest are displayed: glutaminase (GLS) (**f**), ribosomal protein L37 (RPL37) (**g**), and cystathionine gamma-lyase (CTH) (**h**). *n* = 7 patients for single-cell analyses.

As histological classification lacks the ability to examine the metabolic state of different cells within the tumor microenvironment, we performed analyses on a NSCLC scRNA-seq dataset. Using DEGs from LUSC and LUAD identified based on tumor glucose uptake, we extracted genes that fell into seven metabolic super pathways and examined cell-type-specific expression in intratumoral human immune cells (Fig. 6). Notably, glutaminase (GLS) was significantly downregulated in LUAD tumors with high glucose uptake, suggesting a tradeoff between glucose and glutamine metabolism (Fig. 6a). Dimensionality reduction analyses revealed that GLS expression was highest in tumor-infiltrating T cells, suggesting a metabolically flexible phenotype (Fig. 6e, f). In LUAD tumors with high glucose uptake, several metabolic genes had increased expression (Fig. 6c, d). Notably, CTH and RPL37, both involved in amino acid metabolism, were increased, suggesting that glucose provides a proliferative environment in LUAD tumors. When entered into the single-cell dataset, we once again observed T cells having the highest expression of both of these enzymes, further supporting the metabolic flexibility of T cells in NSCLC (Fig. 6g, h).

### Differential protein expression analyses suggest greater genetic driver alterations in LUAD than LUSC based on visceral adiposity and tumor glucose uptake

To examine whether body composition or tumor glucose uptake stratification was related to the degree of genetic driver alterations, we performed exploratory differential protein expression analyses in our patients from The Cancer Protein Atlas (Supplemental Table 2). A larger fraction of proteins were differentially expressed based on VAT and TGU in LUAD than LUSC, but none met our statistical threshold of *p* < 0.001.

## Discussion

Although it is clear that increased tumor SUVmax predicts poor outcomes in lung cancer^31–34^, how systemic metabolism and, specifically, adiposity affects tumor glucose metabolism remains unclear. It appears that overweight and obesity improve the response to immunotherapy in lung cancer^7,8^; however, it is not known whether obesity confers improvements in prognosis in all types of lung cancer, or if this effect is tumor-type specific. In addition, the use of the commonly used and clinically accessible parameter BMI to predict cancer outcomes has come under scrutiny. This is in part because while BMI is tightly correlated with body fat at the population level, it is a poor predictor of adipose tissue mass at the individual level^35,36^. Total body fat mass is a better predictor of cardiometabolic health^36–39^ and cancer risk^40,41^ than BMI. As subcutaneous adipose tissue is generally considered metabolically inert and far less of a cardiometabolic risk factor than visceral and ectopic adipose tissue^42–44^, both the quantity and the location of adipose tissue may be predictive of cancer outcome. Consistent with a tumor type-dependent effect of systemic metabolism to modulate lung cancer initiation and/or progression, patients with non-alcoholic fatty liver disease (i.e., increased ectopic lipid content) were more likely to have non-squamous cell carcinoma^45^. Our findings supported this notion in LUAD, where only visceral fat content was significantly related to tumor glucose uptake, but not subcutaneous fat or BMI.

Of further interest, when the LUSC and LUAD patients with high visceral fat content were compared, the predominant genetic pathways were related to mitochondrial translation and metabolism. Meanwhile, subcutaneous adiposity was more significantly related to immune function, and specifically neutrophil activation. Our combined PET/CT and genomic data suggest that visceral adiposity could be related to tumor metabolic regulation, while subcutaneous adiposity may be more related to immune function. Further, we were unable to rule out the possibility that common genetic driver mutations could influence the immunometabolic pathways, particularly in LUAD.

We determined that patients with high BMI had a favorable prognostic tumor transcriptional environment, while those with high VAT had a detrimental prognostic profile. This evidence supports a recent study that the visceral fat index is an independent predictor of poor survival in patients with NSCLC^46^. Clearly, BMI is not a direct surrogate of metabolic health, nor of adiposity per se.

Both the SUVmax and RNA expression data obtained in this study are consistent with a greater dependence on glucose in LUSC as compared to LUAD. Despite comparable tumor stage, adiposity, and non-tumor tissue glucose uptake, tumor SUVmax in LUSC was twice that of LUAD. Differentially expressed KEGG pathways in tumors of LUSC patients with high vs low tumor glucose uptake were enriched for metabolic pathways, including glucose, pyruvate, fatty acid, and amino acid metabolism, whereas LUAD KEGG analysis provided a less compelling argument for a critical role of regulation of tumor metabolism. In LUAD patients with high glucose uptake, glutaminase expression was decreased, suggesting a tradeoff between glucose and glutamine in LUAD tumors. In the single-cell dataset, we observed the highest expression of glutaminase in tumor-infiltrating T cells as compared to all other major cell types, suggesting interesting regulatory effects related to glucose and glutamine in T cells and tumor cells. However, in LUSC tumors with high glucose uptake, CTH (amino acid metabolism), HSD17B10 (fatty acid oxidation), and RPL37 (protein biosynthesis) were all increased, suggesting that high glucose uptake does in fact reflect high metabolic activity, though of all major macronutrients. These data hint that rationally-derived, precision oncology metabolic adjunct approaches may be more likely to be effective in LUSC as compared to LUAD, and should consider the fact that glucose is likely not the only substrate that contributes to tumor progression in these tumor types.

As NSCLC can be a highly immunogenic tumor type responsive to immunotherapy, it is important to consider how systemic metabolism and body composition may intersect with tumor immune cell infiltration. The fraction of immune cell subtypes may correlate with tumor stage in NSCLC: Zhang et al. demonstrated decreases in the fraction of M0 macrophages and memory B cells in an advanced stage as compared to early-stage LUAD, but no difference in the fraction of immune cell subtypes in an advanced stage as compared to early-stage LUSC^47^. Interestingly, we found that tumor glucose uptake was positively correlated with macrophage content, and negatively correlated with lymphocyte content in LUSC, which is consistent with recent evidence that macrophages are the predominant glucose consumers in the tumor microenvironment^18^. However, in both tumor types, tumor glucose uptake was negatively related to total TIL content. This is consistent with the conventional wisdom that higher SUVmax and lower immune cell content are poor prognostic features. Though it was not possible to correlate single-cell expression with single-cell glucose uptake in human lung tumors because current techniques to assess metabolic flux rates do not have the capacity to perform on a single cell level, our analyses point to tumor-infiltrating T cells as being a hub of metabolic flexibility in NSCLC.

In summary, here we performed a comprehensive analysis of the impact of body composition on human tumor glucose metabolism, transcriptomic landscape, and immune cell infiltration in the two most common subsets of NSCLC. Our data suggest that LUSC is more deeply dependent on glucose than LUAD. Tumor glucose uptake was higher in LUSC than LUAD, and metabolic pathways related to pyruvate, amino acid, and lipid metabolism were all significantly expressed in metabolically active LUSC tumors. In LUSC, all metabolic genes increased in tumors with high glucose uptake, while LUAD hinted at the ability to toggle between glucose and glutamine metabolism. Visceral adiposity was related to metabolic processes between the two tumor types, while subcutaneous adiposity was related to neutrophil and immune tumor pathways. We determined that high BMI was correlated with a beneficial prognostic profile, while high VAT was correlated with a detrimental prognostic tumor profile, providing improved resolution into the “obesity paradox” in lung cancer. Finally, tumor glucose uptake was negatively related to total tumor immune cell content in both tumor types, countering recent evidence that immune cells are the predominant glucose consumers in the tumor microenvironment, and our single-cell dataset suggests that immune cells, and in particular T cells, display the greatest transcriptional metabolic flexibility. This study provides insight into the impact of body composition on transcriptional profiles in LUSC and LUAD, provides evidence for greater metabolic flexibility in LUAD than LUSC tumors, and suggests that adjunctive metabolic precision oncology therapy may be a more promising approach in LUSC. The molecular mechanisms by which BMI and adiposity diverge in their prognosis for lung cancer require further investigation.

## Methods

Utilizing three complementary National Cancer Institute-funded open-source databases, TCIA, TCGA, and the Quantitative Imaging in Pathology (QuIP) with caMicroscope^48,49^, we performed a retrospective cross-sectional analysis to examine phenotypic, metabolic, and genomic intersections of adiposity and tumor metabolism in NSCLC. All patients with lung adenocarcinoma and squamous cell carcinoma, both located in the bronchus and lungs, with an ^18^F-FDG PET/CT scan available from TCIA were studied, and RNA-sequencing and histology were obtained for all subjects for whom this data was available. All subjects provided informed consent in accordance with each site’s institutional review board. Using metabolic genes of interest from our analyses, we validated our findings in a scRNA-seq dataset in patients with NSCLC to examine cell-type-specific metabolic flexibility^50^.

### ^18^F-FDG PET/CT analysis

PET/CT images of TCGA-LUSC^51^ and TCGA-LUAD^52^ were accessed and downloaded via the TCIA Portal. One full-body CT and two PET images (one attenuation corrected and one nonattenuation corrected—when available) were uploaded to the Image J platform using an open-source plug-in PET-CT Viewer, then co-registered and reconstructed^53^. “Any” parameter to select any voxels meeting tissue density criteria of the PET-CT was used as described^9,54,55^.

SUVmax and SUVmean were obtained for tumor and normal tissues including brain, heart, liver, subcutaneous white adipose tissue, and skeletal muscle (deltoid). Tumor SUVmax was corrected to background ^18^F-FDG in the blood, and a tumor-to-descending aorta calculation was made for the tumor SUV.

The volume of visceral, subcutaneous, and total adipose tissue was obtained with two consecutive CT slices between the L3 and L4 vertebrae with a −190 to −30 Hounsfield Unit cutoff^55,56^. Skeletal muscle volume was obtained on the same slices by drawing regions of interest around all major abdominal muscles with a −29 to 50 Hounsfield Unit cutoff^56^. Fat mass and fat-free mass were estimated from the L3 adipose tissue and skeletal muscle mass respectively using previously defined equations^56^.

### RNA-seq expression analyses

Differential expression analysis between LUAD and LUSC was conducted using processed data from XenaBrowser (https://xenabrowser.net; dataset: gene expression RNAseq—IlluminaHiSeq, dataset ID: TCGA.LUAD/LUSC.sampleMap/HiSeqV2, unit: log2(norm_count+1))^57^. This public TCGA expression data was used to identify genes differentially expressed between high VAT, high SubC, and high BMI subgroups in LUAD vs LUSC (*p* < 0.001; median VAT/SubC split). KEGG Gene Sets and GO Processes bar plots presented were obtained using Enrichr (https://maayanlab.cloud/Enrichr/) using DEGs in LUAD vs LUSC^58^. Gene sets shown are all significantly differentially enriched between groups (Adj. *p* < 0.05). Expression heatmaps were generated using the ComplexHeatmap package in R^59^.

Prognostic valuation of genes was obtained from the PRECOG database, with each gene assigned a *Z* score. Genes with *Z* scores >3.09 or <−3.09 were included in analyses as described^60^. Metabolic super pathways for the gene selection to include in single-cell analyses included amino acids, carbohydrates, energy, lipids, nucleotides, vitamin, and the citric acid cycle, selected from the Reactome database (https://reactome.org/)^61^. Single-cell analyses were performed using the Single Cell Portal, and data dimensionality reduction using the Explore feature (https://singlecell.broadinstitute.org/). We selected GSE127465^50^ as it was the only NSCLC dataset currently available for analysis in the Single Cell Explorer tool.

### TIL analyses

TIL data was obtained from a convolutional neural network (CNN) applied to identify patches of cell subtypes, and were confirmed by a pathologist for accuracy^30^. In brief, >5000 histological images from the TCGA database were computationally stained, and the CNN was trained with pathologist categorization of extracted patches. Features were extracted and proportions of individual cell types were quantified from the CNN analyses.

### Proteomics

Proteomics for each of our patients was downloaded from Xenabrowser from The Cancer Protein Atlas and exploratory differential protein expression analyses were performed.

### Statistics

In all comparisons between two groups, Student’s *t* test was performed to compare differences in means. Because most variables followed a normal distribution, Pearson *r*s were calculated in a correlation matrix in Python 3.6, and *p* values were stringently adjusted using Bonferroni’s correction using the Pingouin Python Package. Hypothesis-testing correlations were selected for highlighting in the correlation matrices. Two-tailed *p* values were computed. REMARK reporting guidelines were used where applicable^62^. Statistical significance was determined as *p* values <0.05.

### Ethics

All specimens and images were obtained from patients who have given informed consent from the respective institutional review boards as described in the original publications (LUAD and LUSC from the institutions of the TCGA Network^63,64^, and the single-cell NSCLC dataset from Massachusetts General Hospital^50^).

### Reporting summary

Further information on research design is available in the Nature Research Reporting Summary linked to this article.

## Supplementary information

Supplemental Data

REPORTING SUMMARY

## Data Availability

The imaging, genomics, and histopathology data are publicly available in the TCIA (https://www.cancerimagingarchive.net/), TCGA (https://portal.gdc.cancer.gov/), and caMicroscope (https://wolf.cci.emory.edu//camic_org/apps/landing/landing.html) databases, respectively. Proteomics is available from the TCPA database (https://tcpaportal.org/tcpa/). Prognostic valuation of genes is available from the PRECOG database (https://precog.stanford.edu/index.php). The single-cell RNA-sequencing data are available in the GEO database (GSE127465). Metabolic super pathway gene lists were acquired from Reactome (https://reactome.org/).

## Code availability

Requests for the code used to analyze the above data can be fulfilled by B.P.L. (brooks.leitner@yale.edu) in the form of Jupyter Notebooks.
