# Supplementary material for: Multimodal analysis suggests differential immuno-metabolic crosstalk in lung squamous cell carcinoma and adenocarcinoma
Source: NPJ Precis Oncol. 2022 Jan 27;6:8. doi: 10.1038/s41698-021-00248-2 (PMC8795406; doi:10.1038/s41698-021-00248-2)
Supplement: Supplementary file 1 — Supplemental Data [file 41698_2021_248_MOESM1_ESM.pdf]

Supplemental Table 1. Patient demographics for LUAD and LUSC cohorts. N/A indicates that the data were not available.

		**LUAD (n=18)**	**LUSC (n=17)**
		Mean ± SD	Mean ± SD
**Age (years)**		67.7 + 6.3	70.9 + 8.6
**Sex**	Male	8	10
	Female	9	7
	N/A	1	0
**Body Weight (kg)**		71.2 + 13.1	75.6 + 15.2
**Stage**	I	6	7
	II	1	8
	III	4	2
	IV	1	0
	N/A	6	0
**Race**	Black	2	4
	White	10	13
	N/A	6	0

Supplemental Table 2. Proteomic analyses in patients with LUSC and LUAD, sorted by unadjusted p-value for comparisons between high and low VAT in LUSC and LUAD, along with high and low tumor glucose uptake in LUSC and LUAD.

High vs. Low VAT in LUSC

Protein	low_vat_mean	high_vat_mean	log2FC	T-Statistic	P-Value
DIRAS3	-0.088	0.030	0.119	-3.598	0.009
BAX	0.089	0.564	0.475	-3.253	0.011
EEF2	0.440	-0.144	-0.585	2.847	0.019
PI3KP85	0.328	0.078	-0.250	2.570	0.030
ACC_pS79	-0.039	-0.571	-0.532	2.604	0.031
XBP1	-0.092	-0.394	-0.301	2.513	0.034
VEGFR2	0.151	0.753	0.601	-2.537	0.041
CABL	-0.161	0.081	0.242	-2.299	0.052
CASPASE7CLEAVEDD198	0.635	0.068	-0.567	2.257	0.055
NOTCH1	-0.124	0.187	0.311	-2.172	0.058

High vs. Low VAT in LUAD

Protein	low_vat_mean	high_vat_mean	log2FC	T-Statistic	P-Value
PKCPANBETAII_pS660	0.836	0.106	-0.729	4.093	0.003
GSK3ALPHABETA_pS21S9	0.283	-0.499	-0.782	3.623	0.005
MTOR_pS2448	0.394	-0.113	-0.507	3.950	0.007
RAB11	-0.054	0.309	0.363	-3.382	0.007
X1433ZETA	-0.256	0.106	0.362	-3.533	0.007
X4EBP1	-0.581	-0.096	0.485	-3.386	0.008
INPP4B	0.178	1.180	1.002	-3.362	0.010
S6_pS235S236	0.855	0.000	-0.855	3.351	0.012
GSK3_pS9	0.231	-0.475	-0.706	3.496	0.012
JAK2	0.428	0.026	-0.401	3.044	0.014

High vs. Low Tumor Glucose Uptake in LUSC

Protein	low_tgu_mean	high_tgu_mean	log2FC	T-Statistic	P-Value
TFRC	0.293	1.374	1.082	-3.728	0.006
ASNS	0.041	0.696	0.655	-2.655	0.036
CYCLINE1	-0.505	0.143	0.648	-2.448	0.051
PAXILLIN	0.117	0.571	0.455	-2.271	0.053
PTEN	0.398	-0.174	-0.572	2.423	0.055
BCL2	0.753	-0.092	-0.846	2.232	0.056
P27	0.464	-0.049	-0.512	2.241	0.060
COG3	-0.320	-0.503	-0.183	2.261	0.076
ETS1	-0.491	-0.930	-0.439	2.012	0.079
RAB25	-0.233	-0.648	-0.416	1.952	0.096

High vs. Low Tumor Glucose Uptake in LUAD

Protein	low_tgu_mean	high_tgu_mean	log2FC	T-Statistic	P-Value
X4EBP1	-0.487	0.063	0.551	-4.293	0.002
FOXO3A_pS318S321	0.081	-0.175	-0.256	4.487	0.002
MTOR_pS2448	0.254	-0.222	-0.476	4.336	0.003
GSK3ALPHABETA	-0.202	-0.044	0.158	-4.013	0.004
S6_pS235S236	0.626	-0.192	-0.818	3.420	0.007
ERALPHA	-1.296	-1.822	-0.526	3.741	0.007
X1433ZETA	-0.184	0.223	0.406	-3.373	0.009
MAPK_pT202Y204	0.776	-0.339	-1.115	3.578	0.010
STAT3_pY705	0.773	0.089	-0.684	2.837	0.019
TUBERIN_pT1462	-0.059	-0.297	-0.238	3.061	0.023
