# Supplementary material for: Multimodal analysis suggests differential immuno-metabolic crosstalk in lung squamous cell carcinoma and adenocarcinoma
Source: NPJ Precis Oncol. 2022 Jan 27;6:8. doi: 10.1038/s41698-021-00248-2 (PMC8795406; doi:10.1038/s41698-021-00248-2)
Supplement: Supplementary file 2 — REPORTING SUMMARY [file 41698_2021_248_MOESM2_ESM.pdf]

nature portfolio

Corresponding author(s): Rachel J. Perry and Brooks P. Leitner

Last updated by author(s): Nov 22, 2021

Reporting Summary

Nature Portfolio wishes to improve the reproducibility of the work that we publish. This form provides structure for consistency and transparency in reporting. For further information on Nature Portfolio policies, see our Editorial Policies and the Editorial Policy Checklist.

Statistics

For all statistical analyses, confirm that the following items are present in the figure legend, table legend, main text, or Methods section.

n/a	Confirmed	
☐	☒	The exact sample size (n) for each experimental group/condition, given as a discrete number and unit of measurement
☐	☒	A statement on whether measurements were taken from distinct samples or whether the same sample was measured repeatedly
☐	☒	The statistical test(s) used AND whether they are one- or two-sided<br>*Only common tests should be described solely by name; describe more complex techniques in the Methods section.*
☒	☐	A description of all covariates tested
☐	☒	A description of any assumptions or corrections, such as tests of normality and adjustment for multiple comparisons
☐	☒	A full description of the statistical parameters including central tendency (e.g. means) or other basic estimates (e.g. regression coefficient) AND variation (e.g. standard deviation) or associated estimates of uncertainty (e.g. confidence intervals)
☐	☒	For null hypothesis testing, the test statistic (e.g. F, t, r) with confidence intervals, effect sizes, degrees of freedom and P value noted<br>*Give P values as exact values whenever suitable.*
☒	☐	For Bayesian analysis, information on the choice of priors and Markov chain Monte Carlo settings
☒	☐	For hierarchical and complex designs, identification of the appropriate level for tests and full reporting of outcomes
☒	☐	Estimates of effect sizes (e.g. Cohen's d, Pearson's r), indicating how they were calculated

Our web collection on statistics for biologists contains articles on many of the points above.

Software and code

Policy information about availability of computer code

Data collection	No data was generated or collected for this study.
Data analysis	All data was analyzed using open source Python or R packages, with detailed descriptions in the Methods section.

For manuscripts utilizing custom algorithms or software that are central to the research but not yet described in published literature, software must be made available to editors and reviewers. We strongly encourage code deposition in a community repository (e.g. GitHub). See the Nature Portfolio guidelines for submitting code & software for further information.

Data

Policy information about availability of data

All manuscripts must include a data availability statement. This statement should provide the following information, where applicable:

- Accession codes, unique identifiers, or web links for publicly available datasets
- A description of any restrictions on data availability
- For clinical datasets or third party data, please ensure that the statement adheres to our policy

All URL's and accession codes to the data sources that were used in this manuscript are provided in the Data Availability Statement of the text.

Field-specific reporting

Please select the one below that is the best fit for your research. If you are not sure, read the appropriate sections before making your selection.

☒ Life sciences ☐ Behavioural & social sciences ☐ Ecological, evolutionary & environmental sciences

For a reference copy of the document with all sections, see nature.com/documents/nr-reporting-summary-flat.pdf

Life sciences study design

All studies must disclose on these points even when the disclosure is negative.

Sample size	Sample sizes were chosen based on the full extent of available data from The Cancer Imaging Archive: Ie. all subjects from the TCGA that had PET/CT scans available in the TCIA were included in the analyses, and appropriate statistical corrections (ie. stringent thresholds for statistical significance were applied for the differential gene expression analyses, as well as the correlation analyses in the manuscript).
Data exclusions	No available data was excluded
Replication	No measures were attempted to replicate this data, due to lack of availability of multiple datasets with the complete set of PET/CTs and RNA sequencing.
Randomization	Subjects were divided by median split of different PET and CT parameters (ie. VAT, BMI, and tumor glucose uptake). The authors that performed the PET and CT analyses were unaware at the time of analysis of the gene expression comparisons that were performed.
Blinding	The authors who performed the PET and CT analyses were blinded to the comparisons that were made based on PET and CT subgroups for gene expression analyses. That is AB and SO performed PET and CT analyses, which were used to define the median splits for the patient subsets, while BL and KG performed all gene expression analyses.

Reporting for specific materials, systems and methods

We require information from authors about some types of materials, experimental systems and methods used in many studies. Here, indicate whether each material, system or method listed is relevant to your study. If you are not sure if a list item applies to your research, read the appropriate section before selecting a response.

Materials & experimental systems

n/a	Involved in the study	
☒	☐	Antibodies
☒	☐	Eukaryotic cell lines
☒	☐	Palaeontology and archaeology
☒	☐	Animals and other organisms
☒	☐	Human research participants
☒	☐	Clinical data
☒	☐	Dual use research of concern

Methods

n/a	Involved in the study	
☒	☐	ChIP-seq
☒	☐	Flow cytometry
☒	☐	MRI-based neuroimaging
